# Associations of steps per day and step intensity with the risk of diabetes: the Hispanic Community Health Study / Study of Latinos (HCHS/SOL)

**DOI:** 10.1186/s12966-022-01284-2

**Published:** 2022-04-15

**Authors:** Carmen C. Cuthbertson, Christopher C. Moore, Daniela Sotres-Alvarez, Gerardo Heiss, Carmen R. Isasi, Yasmin Mossavar-Rahmani, Jordan A. Carlson, Linda C. Gallo, Maria M. Llabre, Olga L. Garcia-Bedoya, David Goldsztajn Farelo, Kelly R. Evenson

**Affiliations:** 1 Department of Epidemiology, University of North Carolina at Chapel Hill, 123 W. Franklin St. Suite 410, Chapel Hill, NC 27516 USA; 2 Department of Biostatistics, University of North Carolina at Chapel Hill, Chapel Hill, NC USA; 3 Department of Epidemiology and Population Health, Albert Einstein College of Medicine, Bronx, NY USA; 4 Children’s Mercy Kansas City and University of Missouri Kansas City, Kansas City, MO USA; 5 Department of Psychology, San Diego State University, San Diego, CA USA; 6 Psychology Department, University of Miami, Miami, FL USA; 7 Department of Medicine, University of Illinois at Chicago, Chicago, IL USA; 8 Boston Fusion Corp, Lexington, MA USA

**Keywords:** Steps per day, Step cadence, Diabetes, Physical activity, Hispanic/Latino, Cohort, Epidemiology

## Abstract

**Background:**

Higher levels of moderate-to-vigorous physical activity have been associated with a lower risk of diabetes, but less is known about how daily step counts (steps/day) are associated with diabetes risk. Therefore, we examined the association of steps/day and step intensity with incident diabetes.

**Methods:**

We included 6634 adults from the population-based prospective cohort Hispanic Community Health Study/Study of Latinos (HCHS/SOL) (2008–2017). Cox proportional hazard models that accounted for complex survey design and sampling weights were used to estimate the association of baseline accelerometer-assessed steps/day and step intensity with 6-year risk of incident diabetes as hazard ratios (HR) and 95% confidence intervals (CI). We further examined whether the percent of intense steps at a given accumulation of steps/day was associated with diabetes risk, and if associations were modified by specific cohort characteristics.

**Results:**

The average age of cohort members was 39 years and 52% were female. Adults had an average of 8164 steps/day and spent 12 min/day in brisk ambulation (> 100 steps/min). Over 6 years of follow-up, there were 1115 cases of diabetes. There was a suggestive lower risk of diabetes with more steps/day– adults had a 2% lower risk per 1000 steps/day (HR = 0.98 (95% CI 0.95, 1.00)). Inverse associations between average steps/day and diabetes incidence were observed across many cohort characteristics, but most importantly among adults at high risk for diabetes – those who were older, or had obesity or prediabetes. Adults who accumulated 17 min/day in brisk ambulation compared to < 2 min/day had a 31% lower risk of diabetes (HR = 0.69 (95% CI 0.53, 0.89)). A greater percent of intense steps for a given accumulation of steps/day was associated with further risk reduction.

**Conclusion:**

Adults who accumulate more daily steps may have a lower risk of diabetes. Accumulating more steps/day and greater step intensity appear to be important targets for preventing diabetes.

**Supplementary Information:**

The online version contains supplementary material available at 10.1186/s12966-022-01284-2.

## Background

In the United States (US), over 26 million adults (9.8%) have a diagnosis of type 2 diabetes and 91.8 million (37.6%) have prediabetes [1]. Diabetes is associated with significant morbidity and mortality [2], and will remain a large public health burden, as the prevalence of diabetes is projected to double by 2030 [3]. One preventive strategy advocated by the American Diabetes Association is engagement in moderate-to-vigorous physical activity (MVPA) [4]. Higher levels of MVPA have been associated with a lower risk of diabetes [5–8]. While most evidence relies on self-reported and accelerometer-measured MVPA, less is known about how daily step counts are related to diabetes. Daily step counts are an easily interpretable, trackable, and simple measure of physical activity volume and have become more familiar and accessible to the public with the increase in wearable devices [9]. In 2018, the US Physical Activity Guidelines Advisory Committee reviewed studies on step counts with health outcomes and determined there was insufficient evidence on the association of step counts with mortality, cardiovascular disease, and diabetes [9, 10]. The Committee called for more longitudinal research on the association of step counts and stepping cadence with health outcomes [9]. Since 2018, new studies have been published that suggest greater steps per day are associated with a lower risk of all-cause mortality [11–14]. However, there remains limited data on how daily steps counts are associated with diabetes risk.

Total step volume (steps/day) includes steps accumulated at a light, moderate, and vigorous intensity [9]. The rate of stepping, or cadence (steps/min), is considered a proxy for walking intensity and a directly-observed cadence of > 100 steps/min is suggested to be a moderate intensity activity or greater (≥ 3 metabolic equivalents) [15]. Four longitudinal studies have examined the association of steps/day with diabetes or incident dysglycemia (impaired fasting glucose or impaired glucose tolerance) [16–19]. In the NAVIGATOR Trial, participants had a 4% lower risk of diabetes per 2000 steps/day [16]. In three other cohorts, the Australian Diabetes, Obesity and Lifestyle Study (AusDiab), Healthy Aging Initiative cohort in Northern Sweden, and the Women’s Health Initiative Objective Physical Activity and Cardiovascular Health (OPACH) Study, a lower risk of incident diabetes or dysglycemia was also observed with accumulating more steps/day [17, 18]. These studies focused on steps/day and only one [19] examined how step cadence was associated with diabetes risk. Additionally, it is unclear how well findings from these studies generalize to more diverse populations, especially among population groups such as Hispanic/Latinos who have high rates of diabetes [1]. Furthermore, only the OPACH study [19] of women examined if associations varied by any risk factors for diabetes such as age, obesity, prediabetes, and insulin resistance [1, 2, 20, 21]. Additionally, in populations such as Hispanic/Latinos, occupational physical activity is highly prevalent [22, 23] and findings have been mixed on if occupational physical activity is associated with a lower risk of diabetes [6, 24–26].

Given the limited information on how stepping is associated with diabetes, we examined how steps/day were associated with the risk of diabetes in the Hispanic Community Health Study/Study of Latinos (HCHS/SOL). Furthermore, we examined if specific cohort characteristics (age, sex, occupational physical activity, obesity, insulin resistance, prediabetes, and Hispanic/Latino heritage) modified the association between steps/day and diabetes. Because steps/day is a combination of light, moderate, and vigorous intensity steps, we further examined if step cadence and stepping pattern (bouted stepping) were associated with the risk of diabetes. We further explored the impact of step intensity by examining if achieving the same steps/day at a greater percent of intense steps compared to a lower percent of intense steps was associated with a lower risk of diabetes. Chen et al. [7] observed with the HCHS/SOL cohort a lower risk of diabetes with more minutes spent in MVPA, but the steps/day relationship with diabetes has yet to be examined within the cohort [7].

## Methods

### Study population

The HCHS/SOL is the largest population-based cohort of Hispanic/Latino adults from four US metropolitan areas (Bronx, NY; Chicago, IL; Miami, FL; San Diego, CA) [27, 28]. A total of 16,415 self-identified Hispanic/Latino adults (18–74 years) were recruited and enrolled from randomly selected households (2008–2011) through a multistage area probability design. Visits were conducted in 2008–2011 and 2014–2017 and at both visits, participants had a physical examination and completed questionnaires. Participants are also contacted annually over the phone. The Institutional Review Boards approved the study at each site and all participants gave written informed consent.

### Exposure

At baseline, participants were asked to wear an Actical accelerometer (version B-1, model 198–0200-03) during waking hours for 1 week. The Actical was attached to a belt on the right hip and captured accelerations in 1-min epochs. Non-wear was defined using the Choi algorithm [29]. Participants left the examination visit wearing the accelerometer and were told to undertake their usual activities for the week and to remove the accelerometer only during sleeping, showering, and swimming. The data from 5 AM the day after the examination visit until midnight on day 6 were included in the present analyses to provide a consistent maximum 6-day wear period for all participants. Accelerometer adherence was defined as at least 3 days with 10 h of wear. The 10 h criterion is often used in other studies [30] and 3 of 6 days was selected to represent at least half of the maximum days of wear [31].

Steps were determined by the manufacturer’s step algorithm. The Actical step function has performed well in validation studies, although it may undercount steps at slow walking speeds [32–34]. We averaged steps/day over adherent days and calculated several cadence-based indicators of step intensity, including minutes spent at > 40 steps/min (purposeful steps and faster movement), > 70 steps/min (slow walking and faster movement), and > 100 steps/min (brisk walk and faster movement) [15]. We derived peak 30-min cadence, representing the average cadence of the 30 highest (but not necessarily consecutive) minutes in a day. We examined bouts of consecutive minutes spent at different intensity levels (> 40 steps/min, > 70 steps/min, and > 100 steps/min). A bout was defined as > 10 consecutive minutes above the specified cadence while allowing for interruptions of up to 20% of time below the cadence threshold and less than 5 consecutive minutes below the threshold [35]. The bout also had to start and stop above the cadence threshold [36]. Interruptions allowed for real-life events such as stopping at a traffic light or taking a water break during exercise [35]. We calculated the percent of steps that were at a cadence of > 100 steps/min, termed ‘intense steps’, to examine the contribution of intensity when holding steps/day constant.

### Outcome

We examined two definitions of incident diabetes in order to enhance comparisons of our results with other studies that have used various diabetes definitions. The first definition was based on three criteria: 1) self-reported diagnosis of diabetes, 2) self-reported use of diabetic medication, or 3) laboratory-tested fasting plasma glucose > 126 mg/dl, non-fasting plasma glucose of > 200 mg/dl, 2-h postload oral glucose tolerance test (OGTT) > 200 mg/dl, or glycosylated hemoglobin (HbA1c) > 6.5% [37]. The second diabetes definition was based on self-reported diabetic medication and laboratory values. Self-reported use of diabetic medication and blood samples were collected at Visits 1 and 2. Self-reported diagnosis of diabetes, in addition to collection at Visits 1 and 2, was also collected over eight annual follow-up telephone interviews that occurred during the time between Visits 1 and 2. Prevalent diabetes at baseline was defined using the three criteria definition. One participant had data only for self-reported diagnosis and not for diabetic medications and lab values; therefore, the sample size for diabetes based on two criteria was 6633 rather than 6634. Further details are provided in the Supplemental Methods.

### Covariates

We included the following covariates assessed at baseline in our analysis: age, sex, Hispanic/Latino heritage, HCHS/SOL field center, education, marital status, employment, years lived in the US, self-rated general health, mobility limitations, cigarette packyears, alcoholic drinks per week, energy intake, the 2010 Alternative Healthy Eating Index (AHEI-2010), body mass index (BMI), insulin resistance as measured by homeostasis model assessment of insulin resistance (HOMA-IR), prediabetes, and report of occupational physical activity, with description of the measures in the Supplemental Methods.

### Exclusions

Of the 16,415 enrolled participants, 11,623 attended Visit 2. Of the Visit 2 participants, we excluded those who had diabetes at baseline (*n* = 2541), did not wear the accelerometer at Visit 1 (*n* = 732), were not adherent to accelerometry (adherence was > 3 days with > 10 h of wear; *n* = 1085), experienced an accelerometer malfunction (*n* = 62), and were missing any covariates used in analysis (*n* = 515). We trimmed the top and bottom 1% of the steps/day distribution in order to remove extreme outliers (*n* = 54). After exclusions, the analytic sample consisted of 6634 adults.

### Statistical analysis

The HCHS/SOL study used a stratified two-stage area probability sample design and oversampling occurred at each stage (Supplemental Methods) [28]. All results were adjusted for oversampling of specific population subgroups and for nonresponse at Visit 2 by applying sampling weights (Supplemental Methods). We further adjusted for missing accelerometer data using inverse probability weights (IPW) [38]. The IPWs predicted Actical adherence based on associated variables, as described elsewhere (Supplemental Methods) [31]. The final weight was a product of the sampling weight and IPW. The weights were trimmed and calibrated to the 2010 US Census according to age, sex, and Hispanic/Latino background in the Census block groups of the four HCHS/SOL field centers. Results represent the characteristics of underlying population rather than the cohort participants.

Cox proportional hazard models that accounted for the complex survey design and survey weights were used to estimate the association of step metrics with incident diabetes as hazard ratios (HR) and 95% confidence intervals (CI). Follow-up time was calculated as time from baseline to occurrence of incident diabetes, end of follow-up, or death, whichever occurred first. The time scale for all models was time since baseline. We tested the proportional hazards assumption by examining interactions between each step metric and follow-up time and the assumption was met for all models. We further estimated incidence rates (IR) per 10,000 person-years with Poisson models that accounted for the complex survey design and survey weights. All analyses were conducted with SAS version 9.4 and Stata version 15.0.

We examined continuous and categorical measures of all step metrics. First, we examined the dose-response association of steps/day, peak 30-min cadence, and minutes spent at different cadence with incident diabetes. For each step metric, we tested for non-linearity by specifying models with restricted cubic splines with knots at the 25th, 50th, and 75th percentile of the step metric distribution. For all step metrics, the spline terms were not significant suggesting the relationship between each step metric and diabetes was not curvilinear [39]. In models with step metrics specified as a continuous variable, the 10th percentile of the distribution was used as the referent group. Next, we categorized steps/day based on the graduated step index [40] (< 5000 (sedentary), 5000–7499 (low active), 7500–9999 (somewhat active), 10,000–12,499 (active), and > 12,500 steps/day (highly active)). We classified peak 30-min cadence into four categories [15] (< 60 steps/min (incident, sporadic, and purposeful movement), 60 - < 80 steps/min (slow walking), 80 - < 100 step/min (medium walking), > 100 steps/min (brisk walking and faster ambulation)). We separately examined minutes spent above > 40, > 70, and > 100 steps/min and specified categories of minutes spent at different cadence as quartiles. For minutes spent in bouts, we separately examined bouted minutes above thresholds of > 40, > 70, and > 100 steps/min. We included a category of no bouts and specified remaining categories based on tertiles. For all step metrics we estimated a *p*-value for trend by specifying each step metric as an ordinal variable in models. All models were adjusted for age, a quadratic term for age, sex, Hispanic/Latino background by HCHS/SOL field center specified as a 17-level categorical variable, education, married/partner status, employment status, years in the US, self-rated general health, mobility limitations, cigarette pack years, alcoholic drinks per week, energy intake, AHEI-2010, and accelerometer wear time. We conducted sensitivity analyses by further adjusting for BMI, which we expected to attenuate the association as a mediator.

We further explored the role of intensity by examining if having a greater percent of intense steps at a given steps/day was associated with a lower risk of diabetes than having a lower percent of intense steps at the same steps/day (e.g., 7000 steps/day with 30% versus 10% of these steps at ≥100 steps/min). All comparisons were made to the referent of the 10th percentile of steps/day and percent of intense steps (3400 steps/day and 1%). Last, we examined if the relationship between continuous steps/day and diabetes varied by age (< 50, > 50 years), sex (male, female), occupational physical activity (any, none), obesity (not obese, obese), insulin resistance as measured by HOMA IR (normal, high), prediabetes status (normal, prediabetes), and Hispanic/Latino background (Dominican, Central American, Cuban, Mexican, Puerto Rican, South American, Multi/Other). A likelihood ratio test compared a model with an interaction term between steps/day and the modifier to one without the interaction term and a *p*-value < 0.10 suggested the model with the interaction term was a better fit [41]. Examination of the stratum specific estimates were used to determine if the relationship between steps/day and diabetes varied within each modifier [42, 43]. The modification analysis with occupational physical activity was only among adults who reported working full or part-time (*n* = 3799).

## Results

The mean age of the cohort was 39 years, half were female, 76% were born outside of the 50 US states, and 41% had prediabetes at baseline (Table 1). More than half of the cohort reported being employed and among employed adults, 53% reported any occupational physical activity. The average accelerometer wear time was 15.9 h/day (95% CI 15.7, 16.0). Adults had an average of 8164 steps/day (standard error = 92, median = 7317). On average, adults spent 66, 27, and 12 min/day at a cadence of > 40, > 70, and > 100 steps/min, respectively. Additionally, adults spent 24, 10, and 5 min in bouts at cadences of > 40, > 70, and > 100 steps/min, respectively. The average peak 30-min cadence was 77 steps/min.

**Table 1:** Descriptive characteristics at baseline for HCHS/SOL cohort (2008–2017)

	*N* = 6634	
	% or mean	95% CI
**Age (years), mean**	38.4	(37.8,39.1)
**Center, %**		
Bronx	27.7	(24.6,30.8)
Chicago	15.3	(13.1,17.5)
Miami	30.0	(25.9,34.2)
San Diego	27.0	(23.4,30.6)
**Hispanic/Latino heritage, %**		
Central American	7.8	(6.2,9.3)
Cuban	20.4	(17.2,23.5)
Dominican	9.5	(8.0,11.0)
Mexican	38.4	(35.0,41.8)
Puerto Rican	14.7	(12.9,16.6)
South American	4.9	(4.1,5.7)
Multi/Other	4.3	(3.2,5.3)
**Female, %**	51.6	(49.7,53.4)
**Education, %**		
< high school/no GED	29.0	(27.0,31.0)
high school or GED	28.0	(26.2,29.9)
> high school	43.0	(40.6,45.3)
**Married/partner, %**	48.3	(46.0,50.6)
**Employed, %**	55.4	(53.4,57.4)
**Reported any occupational physical activity**^**a**^**, %**	53.2	(50.0, 56.4)
**Born in continental US, %**	24.1	(22.0,26.3)
**BMI (kg/m**^**2**^**), mean**	28.9	(28.7,29.2)
**HOMA IR, mean**	2.9	(2.8,3.0)
**Have prediabetes, %**	41.0	(39.1,42.9)
**General health, %**		
Excellent/very good	32.4	(30.5,34.4)
Good	46.5	(44.5,48.4)
Fair/poor	21.1	(19.5,22.7)
**Have health/mobility limitation, %**	10.2	(8.9,11.5)
**Cigarette pack years, mean**	4.0	(3.6,4.4)
**Drinks per week, mean**	2.9	(2.6,3.2)
**Energy intake (kcal/day), mean**	2023	(1999,2047)
**Alternative Healthy Eating Index, mean**	47.1	(46.7,47.5)
**Average wear time (hours), mean**	15.9	(15.7,16.0)
**Daily steps, mean**	8164	(7983,8344)
**Peak 30 cadence (steps/min), mean**	77.2	(76.2,78.2)
**Proportion of wear at different cadence, mean**		
0 steps/min	69.0	(68.5,69.5)
1- < 40 steps/min	23.9	(23.5,24.3)
40–99 steps/min	5.8	(5.6,6.0)
> 100 steps/min	1.3	(1.2,1.3)
**Minutes per day spent at different cadence, mean**		
> 40 steps/min	66.4	(64.4,68.3)
> 70 steps/min	27.3	(26.2,28.4)
> 100 steps/min	12.1	(11.4,12.8)
**Minutes per day spent in bouts at different cadence, mean**		
> 40 steps/min	24.3	(22.9,25.7)
> 70 steps/min	10.2	(9.5,11.0)
> 100 steps/min	4.8	(4.4,5.2)
**Percent of steps** **>** **100 steps/min, mean**	13.5	(12.9, 14.2)

*Abbreviations BMI* body mass index, *HOMA IR* homeostasis model assessment of insulin resistance

^a^Among participants who were employed (*n* = 3799)

### Step volume with diabetes

Over a median of 5.9 years (range 1 to 9.6 years) of follow-up, there were 1115 (12.5%) and 740 (8.2%) incident events of diabetes based on the two diabetes definitions. Generally, incidence rates and HRs were lower with greater steps/day; however, in the majority of analyses, the confidence intervals included the null value (Table 2). For diabetes based on three criteria, the risk of diabetes was 2% lower (HR = 0.98 (95% CI 0.95, 1.00)) per 1000 steps/day. The predicted risk of diabetes at 7000 and 10,000 steps/day was 8% (HR = 0.92 (95% CI 0.85, 1.01)) and 13% (HR = 0.87 (95% CI 0.74, 1.02)) lower compared to 3400 steps/day (Supplemental Table 1). Analyses with steps/day based on the graduated step index suggested an 18% lower risk of diabetes at 10,000–12,500 steps/day (HR = 0.82 (95% CI 0.57, 1.18) compared to < 5000 steps/day. All results were similar for diabetes based on two criteria. For both diabetes definitions, the addition of BMI to models attenuated all associations of steps/day with the risk of diabetes (Supplemental Table 2).

**Table 2:** The association of average daily steps and the graduated step index with incident diabetes, HCHS/SOL cohort (2008–2017)

	diabetes based on self-reported diagnosis, medications, labs (3 criteria definition) *n* = 6634				diabetes based on medications and labs (2 criteria definition) *n* = 6633			
	num events	sum p-years	adjusted IR per 10,000 p-years	HR (95% CI)	num events	sum p-years	adjusted IR per 10,000 p-years	HR (95% CI)
Steps per day								
< 5000	323	10,049	9.5 (2.0, 44.4)	ref	223	10,519	3.4 (0.5, 23.1)	ref
5000 - < 7500	303	10,263	8.9 (1.9, 41.1)	0.94(0.73,1.22)	190	10,712	2.8 (0.4, 18.7)	0.81(0.60,1.10)
7500 - < 10,000	237	7843	9.9 (2.1, 47.7)	1.04(0.80,1.35)	158	8147	3.5 (0.5, 24.2)	1.01(0.72,1.41)
10,000 - < 12,500	116	4870	7.9 (1.6, 38.8)	0.82(0.57,1.18)	78	5056	2.2 (0.3, 14.9)	**0.60(0.41,0.87)**
> 12,500	136	5281	7.7 (1.6, 37.3)	0.81(0.58,1.14)	91	5464	2.3 (0.3, 16.3)	0.69(0.46,1.02)
p for trend				0.211				**0.032**
per 1000 steps				0.98(0.95,1.00)				0.97(0.94,1.00)

Models adjusted for age (continuous), quadratic term for age, sex (male, female), Latino background by HCHS/SOL field center (17 level categorical variable), education (< high school/no GED, high school/GED, > high school), married/partner status (yes, no), employment (yes, no), years in the US (born in US, < 10 years, > 10 years), self-rated general health (excellent/very good, good, fair/poor), mobility limitations (yes, no), cigarette pack years (continuous), alcoholic drinks per week (continuous), energy intake (continuous), AHEI-2010 (continuous) and accelerometer wear time (continuous, hours per day)

*Abbreviations*: *CI* confidence interval, *HR* hazard ratio, *IR* incidence rate, *p-years* person-years

### Interactions of steps/day and modifiers with diabetes

**Fig. 1:**
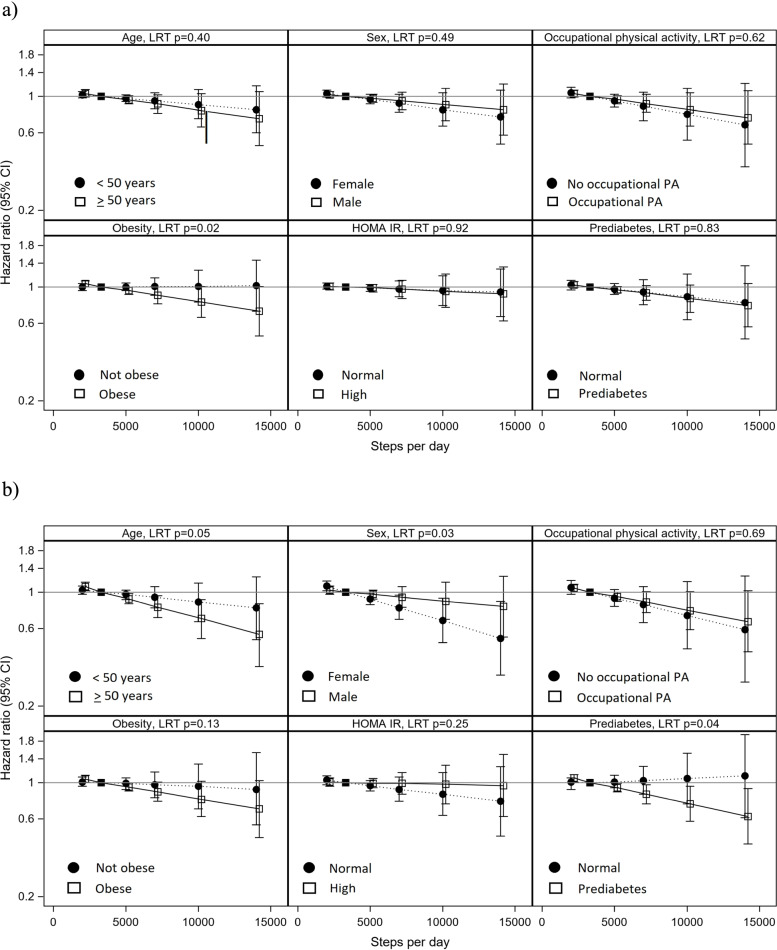
Hazard ratios and 95% CI of the association of steps/day with incident diabetes by modifying factors, HCHS/SOL cohort (2008–2017). **a** Diabetes based on self-reported diagnosis, medications, labs (3 criteria definition, *n* = 6634). **b** Diabetes based on medications and labs (2 criteria definition) *n* = 6633. Predicted estimates at the 2nd percentile (2000 steps/day), 25th percentile (5000 steps/day), 50th percentile (7000 steps/day), 75th percentile (10,000 steps/day), and 90th percentile (14,000 steps/day). Estimates compared to the 10th percentile (3400 steps/day) of steps/day. Abbreviations: CI = confidence interval, HOMA IR = homeostasis model assessment of insulin resistance, LRT = likelihood ratio test, PA = physical activity. All models adjusted for age (continuous), quadratic term for age, sex (male, female), Latino background by HCHS/SOL field center (17 level categorical variable), education (< high school/no GED, high school/GED, > high school), married/partner status (yes, no), employment (yes, no), years in the US (born in US, < 10 years, > 10 years), self-rated general health (excellent/very good, good, fair/poor), mobility limitations (yes, no), cigarette pack years (continuous), alcoholic drinks per week (continuous), energy intake (continuous), AHEI-2010 (continuous) and accelerometer wear . The model with occupational physical activity as a modifier is only among those who reported part- or full-time employment (*n* = 3799) and does not include a covariate for employment, but otherwise is adjusted for the same covariates as other models

For diabetes classified by three criteria, we observed modification by obesity (Fig. 1a, Supplemental Tables 3–4). Adults with obesity had higher incidence rates of diabetes than adults without obesity but adults with obesity who accumulated 10,000 steps/day had 0.81 (95% CI 0.65,1.01) times the risk of diabetes compared to adults with obesity who accumulated 3400 steps/day, while there was no association among adults without obesity (HR = 1.01 (95% CI 0.81, 1.27)).

For diabetes classified by two criteria, we observed modification by age, sex, and prediabetes (Fig. 1b, Supplemental Tables 5–6). Among adults 50 and older, those who accumulated 10,000 steps/day had 0.69(95% CI 0.52,0.90) times the risk of diabetes compared to those who accumulated 3400 steps/day, while for younger adults the inverse association was weaker (HR = 0.87 (95% CI 0.66, 1.14)). Among women, the HR at 10,000 steps/day was 0.67 (95% CI 0.49,0.92) compared to women who accumulated 3400 steps/day while for men there was a weaker inverse association (HR = 0.88 (95% CI 0.68, 1.15)). Adults with prediabetes had higher incidence rates of diabetes than adults without prediabetes, but adults with prediabetes who accumulated 10,000 steps/day had a 26% (HR = 0.74(95% CI 0.58,0.95)) lower risk of diabetes than adults with prediabetes who accumulated 3400 steps/day, while there was no association among adults without prediabetes (HR = 1.06 (95% CI 0.74, 1.52)). No modification was observed by occupational physical, HOMA IR, and Hispanic/Latino heritage.

### Step cadence with diabetes

For both diabetes definitions, spending more time at > 70 and > 100 steps/min and having a faster peak 30-min cadence were associated with a lower risk of diabetes (Table 3). For example, accumulating at least 17 min/day at > 100 steps/min was associated with a 31% (HR = 0.69 (95% CI 0.53,0.89)) lower risk of diabetes (three criteria definition) compared to those who accumulated less than 2 min/day. For both diabetes definitions, the HR estimates were generally lower with more time spent in bouts at each cadence but the confidence intervals included the null value for all analyses (Supplemental Table 7).

**Table 3:** The association of step cadence with incident diabetes, HCHS/SOL cohort (2008–2017)

Step metric - per day	diabetes based on self-reported diagnosis, medications, labs (3 criteria definition) *n* = 6634		diabetes based on medications and labs (2 criteria definition) *n* = 6633	
	adjusted IR per 10,000 p-years	HR (95% CI)	adjusted IR per 10,000 p-years	HR (95% CI)
**Peak 30 cadence, step/min**				
< 60	9.5 (2.1, 44.0)	ref	3.3 (0.5, 23.3)	ref
60 - < 80	8.7 (1.9, 40.0)	0.90(0.70,1.15)	2.9 (0.4, 19.6)	0.86(0.64,1.16)
80 - < 100	7.8 (1.7, 36.3)	0.79(0.62,1.02)	2.6 (0.4, 18.5)	0.75(0.55,1.02)
> 100	5.6 (1.2, 26.3)	**0.58(0.41,0.82)**	1.8 (0.2, 12.9)	**0.56(0.35,0.89)**
p for trend		**0.001**		**0.006**
per 10 step/min increase		**0.95(0.91,0.99)**		**0.93(0.88,0.98)**
**Minutes spent at different cadence**				
*>* *40 steps/min, min per day*				
< 33	9.2 (2.0, 42.9)	ref	3.0 (0.4, 20.6)	ref
33 - < 55	9.3 (2.0, 43.4)	1.03(0.79,1.33)	2.9 (0.4, 19.8)	0.98(0.73,1.33)
55 - < 87	8.5 (1.8, 40.4)	0.91(0.71,1.17)	2.8 (0.4, 18.8)	0.89(0.65,1.21)
> 87	7.4 (1.5, 36.0)	0.78(0.57,1.07)	2.0 (0.3, 13.3)	**0.61(0.43,0.86)**
p for trend		0.092		**0.007**
per 10 min		0.98(0.96,1.00)		0.97(0.94,1.00)
*>* *70 steps/min, min per day*				
< 10	9.8 (2.1, 45.0)	ref	3.2 (0.5, 22.4)	ref
10 - < 21	8.9 (2.0, 40.9)	0.92(0.71,1.18)	3.1 (0.5, 21.4)	0.99(0.73,1.34)
21 - < 39	9.3 (2.0, 43.6)	0.96(0.75,1.23)	2.9 (0.4, 20.4)	0.88(0.66,1.17)
> 39	6.7 (1.5, 30.7)	**0.68(0.51,0.91)**	2.1 (0.3, 14.6)	**0.65(0.45,0.94)**
p for trend		**0.024**		**0.016**
per 10 min		0.95(0.90,1.00)		**0.93(0.87,0.99)**
*>* *100 steps/min, min per day*				
< 2	9.8 (2.1, 44.9)	ref	3.5 (0.5, 24.2)	ref
2 - < 7	9.3 (2.0, 43.3)	0.92(0.71,1.20)	3.0 (0.5, 20.4)	0.83(0.61,1.13)
7 - < 17	8.8 (1.9, 40.4)	0.90(0.71,1.15)	3.2 (0.5, 21.9)	0.97(0.73,1.29)
> 17	6.8 (1.5, 30.9)	**0.69(0.53,0.89)**	2.1 (0.3, 14.6)	**0.64(0.47,0.89)**
p for trend		**0.006**		**0.018**
per 10 min		0.93(0.86,1.00)		0.92(0.83,1.02)
**Percent of steps at** **>** **100 steps/min, %**				
per 10%		0.94(0.87,1.01)		0.95(0.86,1.04)

Models adjusted for age (continuous), quadratic term for age, sex (male, female), Latino background by HCHS/SOL field center (17 level categorical variable), education (< high school/no GED, high school/GED, > high school), married/partner status (yes, no), employment (yes, no), years in the US (born in US, < 10 years, > 10 years), self-rated general health (excellent/very good, good, fair/poor), mobility limitations (yes, no), cigarette pack years (continuous), alcoholic drinks per week (continuous), energy intake (continuous), AHEI-2010 (continuous) and accelerometer wear time (continuous, hours per day)

*Abbreviations*: *CI* confidence interval, *HR* hazard ratio, *IR* incidence rate, *p-years* person-years

For both diabetes definitions, the addition of BMI to models attenuated all associations of cadence-based metrics with the risk of diabetes (Supplemental Table 2).

### Percentage of intense steps at a given step volume

**Fig. 2:**
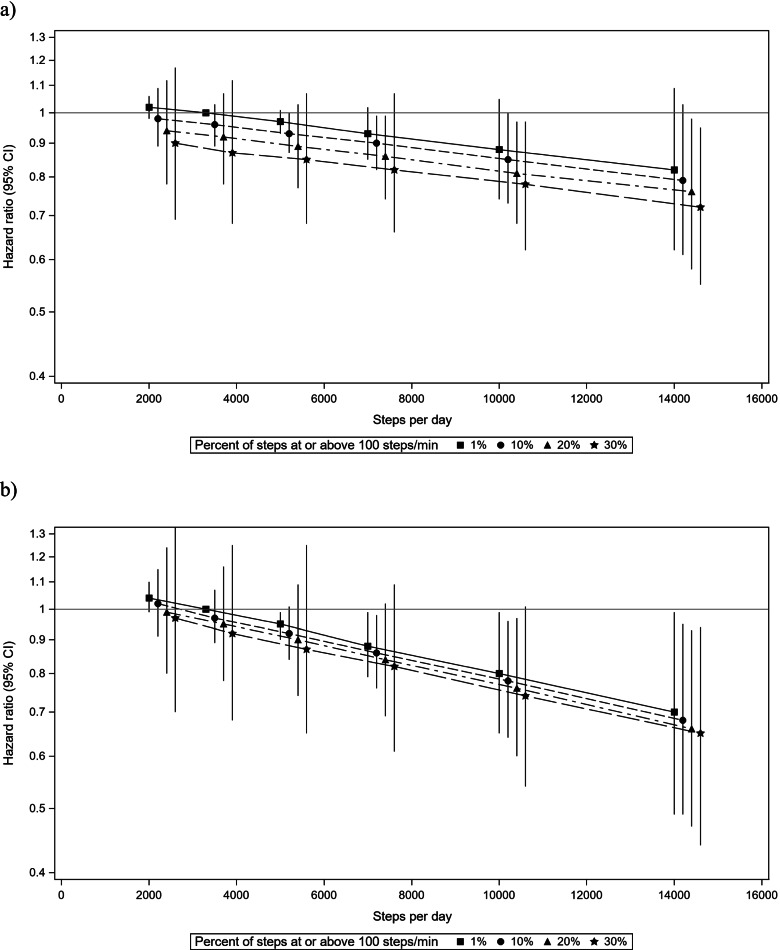
The association of steps/day and percent of intense steps (> 100 steps/min) with incident diabetes, HCHS/SOL cohort (2008–2017). Compared to adults who accumulated 3400 steps/day and had 1% of intense steps (referent), a lower risk of diabetes was observed with greater steps/day, and at a given steps/day, a lower risk was found with greater percent of intense steps. **a** Diabetes based on self-reported diagnosis, medications, labs (3 criteria definition, *n* = 6634). **b** Diabetes based on medications and labs (2 criteria definition) *n* = 6633. Abbreviations: CI = confidence interval. Steps/day predicted estimates at the 2nd percentile (2000 steps/day), 25th percentile (5000 steps/day), 50th percentile (7000 steps/day), 75th percentile (10,000 steps/day), and 90th percentile (14,000 steps/day). Percent of intense steps (> 100 steps/min) predicted estimates at 50th percentile (10%), 75th percentile (20%), and 90th percentile (30%). All comparisons made to the referent of the 10th percentile of steps/day and percent of intense steps (3400 steps/day and 1%). Models adjusted for age (continuous), quadratic term for age, sex (male, female), Latino background by HCHS/SOL field center (17 level categorical variable), education (< high school/no GED, high school/GED, > high school), married/partner status (yes, no), employment (yes, no), years in the US (born in US, < 10 years, > 10 years), self-rated general health (excellent/very good, good, fair/poor), mobility limitations (yes, no), cigarette pack years (continuous), alcoholic drinks per week (continuous), energy intake (continuous), AHEI-2010 (continuous) and accelerometer wear time (continuous, hours per day)

Lower risk of diabetes was observed with greater steps/day and a greater percent of intense steps at a given steps/day level (Fig. 2). Compared to adults who accumulated 3400 steps/day and had 1% of intense steps, adults who accumulated 7000 steps/day and had 10% of intense steps had a 10% (HR = 0.90 (95% CI 0.82, 0.99)) lower risk, whereas adults who accumulated the same steps/day but had 30% of intense steps had a 18% (HR = 0.82 (95% CI 0.66, 1.02) lower risk of diabetes (three criteria definition) (Supplemental Table 8). Findings were similar for diabetes based on two criteria.

## Discussion

In this cohort of Hispanic/Latino adults, we observed that taking more steps/day and spending more time at a faster cadence were associated with a lower risk of developing diabetes. Our results suggest that adults had about a 2 to 3% lower risk of diabetes per 1000 steps/day over 6 years. Our dose-response analysis suggested a gradual decline in risk with more steps/day, such that any amount of stepping was associated with a lower risk of diabetes but greater risk reduction was achieved by taking more steps/day. A faster peak 30-min cadence and more time at faster cadences were also associated with a lower diabetes risk. Further, our results suggested that accumulating the same steps/day at a greater percent of intense steps provided greater risk reduction than reaching the same steps/day level with a lower percent of intense steps. Thus, both volume and intensity may be important for lowering the risk of diabetes. We found stronger associations between steps/day and reduced diabetes incidence among adults at higher risk for diabetes, including older adults and those affected by obesity and prediabetes.

Our inverse association between steps/day and diabetes is similar to findings from the NAVIGATOR Trial where diabetes was defined by fasting glucose and 2-h OGTT. In the NAVIGATOR study, 9306 participants with impaired glucose tolerance, had a 4% (HR = 0.96 (95% CI 0.94, 0.99)) lower risk of diabetes per 2000 steps/day (2% per 1000 steps/day) [16]. With diabetes defined by medications and labs, we estimated a 3% lower risk per 1000 steps (HR = 0.97 (95% CI 0.94,1.00)). The OPACH study estimated a HR of 0.88 (95% CI 0.78, 1.00) between steps/day and diabetes defined by self-report of physician diagnosed diabetes requiring the need of insulin or hypoglycemic medication [19]. The AusDiab study also examined steps/day and diabetes risk and observed a 13% lower risk per 1000 steps/day of incident dysglycemia over 5 years, but only two participants developed diabetes [17]. In a recent analysis with 3055 Swedish adults of the Healthy Aging Initiative study, the authors observed a 59% (HR = 0.41 (95% CI 0.25, 0.66) lower risk of diabetes, defined by International Classification of Disease codes, for accumulating > 4500 steps/day compared to < 4500 steps/day [18]. Although our HR estimates suggest a lower diabetes risk with greater steps/day, the confidence intervals for many estimates did include the null value.

In addition to these studies on steps/day, there has been consistent evidence on the inverse association of self-reported physical activity with diabetes risk, but more evidence is needed on the dose response relationship [5, 6]. A recent meta-analysis on self-reported physical activity and diabetes risk observed that total physical activity, as well as many subtypes of physical activity (leisure-time activity, low, moderate, and vigorous intensity activity, walking, occupational activity, and resistance exercise) were associated with a lower risk of diabetes [6]. In the meta-analysis the dose-response relationship of leisure-time activity with diabetes was curvilinear, but there were too few studies to examine the total physical activity dose response relationship with diabetes [6]. In our analysis with HCHS/SOL, total physical activity volume measured as steps/day suggested a linear relationship with diabetes. A similar linear relationship was observed with the OPACH study between steps/day and risk of diabetes [19]. With the HCHS/SOL study, Chen et al. [7] examined accelerometer measured minutes in MPVA and observed a curvilinear relationship with diabetes risk. We further investigated this finding by examining the correlation between steps/day and minutes in MVPA and found a correlation of 0.66 (Supplemental Table 9). It is possible that the large amount of lower intensity activity that makes up the step/day distribution may account for the different dose response relationship between MVPA and steps/day with diabetes risk. However, further studies should examine the shape of the relationship between objectively measured total physical activity volume, steps, and MVPA with incidence of diabetes.

It is hypothesized that physical activity may lower the risk of diabetes through several biological mechanisms. Greater amounts of physical activity may reduce adiposity, a risk factor for diabetes [6]. Additionally, both acute and long-term physical activity increase glucose uptake in skeletal muscle cells [44]. Muscle contractions, independent of insulin, increase glucose transport from blood into skeletal muscle by translocation of the glucose transporters, especially glucose transporter 4 (GLUT4), from the intercellular location to the plasma membrane [45]. Long-term physical activity is associated with adaptations to skeletal muscle, including an increase in GLUT4 protein levels and mitochondrial enzyme content, and alteration of muscle fiber types that promote glucose transport [44]. These mechanisms lend support to the evidence that greater amounts of physical activity are associated with a lower risk of diabetes.

Diabetes risk is higher among older age groups and those with prediabetes or obesity [2, 20]. We found a stronger inverse association between steps/day and diabetes risk for adults 50 years and older, and those affected by obesity or prediabetes. In a previous HCHS/SOL analysis, a lower risk of diabetes was also observed with more accelerometer measured minutes spent in MVPA among adults older than 50 years supporting this finding [7]. Exercise interventions among adults with obesity and those with impaired fasting glucose have consistently documented a lower diabetes risk with participation in physical activity and diet interventions [46–48] and many of these trials have noted stronger risk reduction among older than younger adults [48, 49]. Our results suggest that adults at higher risk for diabetes, such as older adults and those with prediabetes and obesity, should be encouraged to engage in more steps/day to lower their risk. A stronger inverse association between steps/day and the risk of diabetes was observed for women than men. It is possible that other risk factors, such as hypercholesteremia that are more prevalent among men than women in HCHS/SOL [50], may have may elevated diabetes risk more among men.

Our results regarding cadence, a proxy for step intensity, suggest that a higher peak 30-min cadence and more time at a faster cadence were associated with a lower risk of diabetes. Peak 30-min cadence has been described as a summary of an individual’s best natural effort [15]. Adults with an average peak 30-min cadence of ≥100 steps/min, which is a cadence described as a brisk walk or faster ambulation [15], had about a 40% lower risk of diabetes compared to adults who had an average peak 30-min cadence of less than 60 steps/min. Additionally, adults who accumulated > 39 min and > 17 min at > 70 and > 100 steps/min respectively, cadences described as slow and brisk walking [15], were associated with about a 30% lower risk of diabetes compared to adults who accumulated less time at each of these cadences. The OPACH study examined peak 30-min cadence and percent of time at > 40 steps/min and did not find strong associations between these cadence measures and the risk of diabetes [19]. The OPACH study also classified steps of light and moderate-to-vigorous intensity using vector magnitude counts and observed a HR of 0.86 (95% CI 0.74, 1.00) between moderate-to-vigorous steps (per 2000 step increment) and diabetes risk, but no association between light steps and diabetes [19]. Two cross-sectional studies [51, 52] with the 2005–2006 National Health and Nutrition Examination Survey data reported that greater peak 30-min cadence was associated with improved cardiometabolic risk factors. We observed that more time spent in stepping bouts was generally associated with a lower risk of diabetes, however the confidence intervals included the null value in all analyses. The OPACH study found that steps that were accumulated in bouts of 5 min or more were not associated with diabetes risk [19]. Few studies reported on bouted stepping and diabetes risk, but others have examined accelerometer-assessed MVPA bouts with various health outcomes and found that total volume of physical activity was more important than the pattern of activity [53, 54].

In addition to observing a potential lower risk of diabetes with more steps/day, our results suggest that the risk of diabetes was even lower when these steps were accumulated at a higher intensity. Adults who accumulated 7000 steps/day with 10% versus 30% being intense steps (≥100 steps/min) had a 10% versus 18% lower risk of diabetes, respectively, compared to those accumulating 3400 steps/day with 1% being intense steps. At 7000 steps/day, 10 and 30% of intense steps are equivalent to accumulating 700 and 2100 steps at a brisk walk or faster pace which can be reached by taking a 7 min and 21 min brisk walk. In support of our finding, Strain et al. [55], in the UK Biobank study, observed a lower risk of mortality with greater physical activity volume and that accumulating the same volume with more intense activity rather than lower intensity activity had even greater benefits. Our study suggests that greater step intensity at a given step volume is associated with a further risk reduction of diabetes.

A strength of our study is that we focused on steps/day, an easy-to-understand metric [9] that has become more accessible due to increased activity tracker and smartphone usage [56], and can motivate adults to increase their physical activity [57]. Use of pedometers and activity trackers to monitor steps/day have been an effective strategy to increase daily steps/day [57, 58]. The common 10,000 steps/day goal present on many activity trackers was not based on scientific evidence but developed from the name of a Japanese pedometer [59]. Currently, there are no guidelines that provide recommendations on the number of steps/day needed to achieve optimal health outcomes [9]. Some research suggests that 7000–8000 steps/day are necessary to meet the aerobic physical activity guidelines based on minutes spent in MVPA [60]. Research on steps/day and health outcomes is quickly growing [11, 61] and our findings can contribute to future efforts to develop evidence-based steps/day guidelines.

Our analysis is unique, as it was conducted with the largest longitudinal Hispanic/Latino cohort. The Hispanic/Latino population accounts for 18% (60 million) of the US population [62] and is projected to grow to 28% by 2060 [63]. A strength of our study is that we used the Actical accelerometer to measure steps and step cadence, rather than relying on self-report of steps from pedometers. Because of the Actical accelerometer, which provided time-stamped data on step accumulation, we were able to capture indicators of step intensity and bouts, which has enabled further understanding of how stepping patterns are associated with the risk of diabetes. Additionally, we used two definitions of diabetes and generally observed similar results for both definitions.

There are limitations to our analysis to be considered. Reverse causality cannot be ruled out as we are unable to determine exactly when diabetes developed and adults who developed diabetes close to baseline may have lower steps/day. We used one measure of steps/day and cadence and acknowledge that activity patterns may change over time. However, data from the Women’s Health Study examined steps/day over 3 years and found that a 7-day accelerometer collection of steps/day was a reasonable estimate of longer-term physical activity [64]. Steps/day only captures ambulatory movement and not non-ambulatory movement, such as any upper body movement while standing still, or activities such as cycling or swimming. It is possible that non-ambulatory movement may be beneficial for preventing diabetes in addition to ambulatory movement. We examined if participants who were included in our analysis were healthier than those excluded and observed few differences in demographic and health characteristics between the two groups. Additionally, the sampling weights accounted for attrition and differences in accelerometer wear and adherence.

## Conclusion

In conclusion, engaging in more steps/day and taking more steps at a brisk walk or faster pace is potentially associated with a lower risk of diabetes among US Hispanic/Latino adults. Our results suggest that adults had a 2 to 3% lower 6-year risk of diabetes per 1000 steps/day -- any amount of stepping was associated with a lower risk of diabetes but greater risk reduction was achieved by taking more steps/day. The inverse association between steps/day and diabetes risk was observed across many cohort characteristics, most prominently among adults at high risk for diabetes – those who were older, or had obesity or prediabetes. Accumulating the same steps/day at a greater percent of intense steps was associated with further risk reduction. Our results suggest adults can lower their risk of diabetes by taking more steps/day at any pace, but should engage in brisk walking for part of their day to gain the greatest benefit.

## Supplementary Information

**Additional file 1.**

## Data Availability

HCHS/SOL study data can be accessed by contacting the Collaborative Studies Coordinating Center at the University of North Carolina at Chapel Hill at the following email address: HCHSAdminstration@unc.edu.

## Abbreviations

AHEI: Alternative Healthy Eating Index
BMI: Body Mass Index
CI: Confidence Interval
HbA1c: Glycosylated hemoglobin
HCHS/SOL: Hispanic Community Health Study/ Study of Latinos
HOMA-IR: Homeostasis model assessment of insulin resistance
HR: Hazard Ratio
IPW: Inverse Probability Weights
MVPA: Moderate-to-vigorous physical activity
OGTT: Oral glucose tolerance test
