# Supplementary material for: Associations of steps per day and step intensity with the risk of diabetes: the Hispanic Community Health Study / Study of Latinos (HCHS/SOL)
Source: Int J Behav Nutr Phys Act. 2022 Apr 15;19:46. doi: 10.1186/s12966-022-01284-2 (PMC9013106; doi:10.1186/s12966-022-01284-2)
Supplement: Supplementary file 1 — Additional file 1. [file 12966_2022_1284_MOESM1_ESM.docx]

**Supplemental Methods**

Diabetes Outcome

Participants were asked to fast for at least 8 hours and refrain from smoking the day of their examination visit. Plasma glucose was assayed with the hexokinase enzymatic method (Roche diagnostics Corporation, Indianapolis, IN). Glycosylated hemoglobin was measured in EDTA whole blood with a Tosoh G7 automated high-performance liquid chromatography analyzer (Tosh Bioscience Inc., San Francisco, CA).(1) Participants who had a fasting glucose <150 mg/dl (measured with a glucose meter during the examination visit) and no self-reported history of diabetes completed a 2-hour OGTT with a 75-gram glucose load.(1)

Covariates

Participants self-identified as Central American, Cuban, Dominican, Mexican, Puerto Rican, South American, or Multi/Other. During the interview participants reported their highest level of educational attainment, their marital/partner status, if they were currently employed part-time or full time or not working/retired, and rated their health. Participants reported if they were born in the 50 US states, or if born outside of the US, then they reported the number of years they had lived in the US.

Mobility limitation was ascertained by two questions from the SF-12 (version 2) questionnaire. Participants who reported their health limited them a lot to perform moderate activities (such as moving a table, vacuuming, bowling or playing golf) or to climb stairs were coded as having limitations, while those who answered they were not limited a lot to both questions were coded as not having limitations. Cigarette pack years was constructed by multiplying the number of years a participant reported smoking by the average number of cigarettes smoked per day and then divided by 20 (the number of cigarettes in a pack). Alcoholic drinks per week was created by summing the number of alcoholic drinks (wine, beer, liquor, spirits, or mixed drinks) that participants reported consuming in a week.

Dietary data were collected with two 24-hour dietary recalls at baseline. Dietary energy was computed based on the National Cancer Institute (NCI) methodology.(2) The 2010 Alternative Healthy Eating Index (AHEI-2010) assessed diet quality and was based on 11 dietary components (vegetables, fruit, whole grains, sugar-sweetened beverages, nuts and legumes, red/processed meat, trans fat, long-chain fats, polyunsaturated fatty acids, sodium, and alcohol). For each component, the NCI method was used to predict usual intake. Scores were assigned to each component based on recommended intake. The 11 components were summed and the overall score ranged from 0 (nonadherence) to 110 (perfect adherence).(3, 4)

Weight and height were measured during the examination visit by trained and certified staff and body mass index (BMI) was calculated as weight in kilograms divided by height in squared meters. Obesity was defined as a BMI > 30 kg/m^2^. Insulin resistance was quantified based on the homeostasis model assessment of insulin resistance (HOMA-IR) and was calculated as fasting glucose multiplied by fasting insulin and divided by 405. We categorized insulin resistance as a HOMA-IR > 75^th^ percentile of the HCHS/SOL sample, which was a HOMA-IR of 3.5. Participants were considered prediabetic if they had a fasting plasma glucose of 100-125 mg/dl, or OGTT of 140 - <200 mg/dl, or HbA1c of 5.7% - < 6.5%.

Self-reported occupational physical activity was collected with a modified Global Physical Activity Questionnaire (GPAQ)(5) that has good reliability and validity.(6, 7) We included physical activity in the past week that occurred at work. Participants were asked to think about physical activity that occurred at work for at least 10 minutes continuously and to report the number of days per week and hour and minutes per day spent in moderate and vigorous intensity activities.(8) All questionnaires in which covariates were ascertained can be accessed at the HCHS/SOL study website.(9)

Statistical Analysis

The HCHS/SOL study used a stratified two-stage area probability sample design.(10) The first sampling stage randomly selected census block groups based on Hispanic/Latino concentration and proportion of high/low socioeconomic status in each of the four HCHS/SOL sites.(10, 11) The second stage was a random sample of households selected from lists of postal addresses from the selected census block groups. Oversampling occurred at each stage to increase the likelihood that a selected address would yield an eligible household.(10) As a result of the oversampling, participants were selected with unequal probabilities of selection and these probabilities need to be taken into account during analysis.(11)

Sampling weights reflect the unequal probabilities of selection at each stage. The sampling weights are the product of a base weight (reciprocal of the probability of selection) and three multiplicative adjustments including nonresponse relative to the sampling frame, trimming to reduce the variability of the weights and the impact of extremely large weights values, and calibration to the 2010 US Census according to age, sex, and Hispanic/Latino background.(11) Weights for Visit 2 were calculated based on the sampling weights for Visit 1 and participant nonresponse at Visit 2. A classification tree approach (R package rpart) was used to identify baseline factors associated with attending Visit 2.(12) The factors that were predictive of attending Visit 2 included annual follow-up exam refusal, distance to field center, Hispanic/Latino background, eGFR, sex, strata, and education. The Visit 2 sampling weight was calculated based on the Visit 1 nonresponse sampling weight and the nonresponse rates for Visit 2. Then the Visit 2 sampling weight was trimmed and calibrated to the age, sex, and Hispanic/Latino background of 2010 US Census.

Inverse probability weights (IPW) were used to account for missing accelerometer data.(13) First, multiple imputation was used to impute missing covariates. Then, for each of five imputed datasets, logistic regression was used to predict accelerometer adherence using the following covariates: age, sex, income, marital status, education, employment status, language preference, immigrant generation, self-reported physical activity, BMI, and physical health.(14) Next, the five linear predictors (one from each imputed dataset) were averaged to calculate the probability of being adherent. The final weight used in our analysis was the multiplicative product of the inverse of the probability of being adherent (IPW) with the Visit 2 sampling weight.

**References**

1. Schneiderman N, Llabre M, Cowie CC, Barnhart J, Carnethon M, Gallo LC, et al. Prevalence of diabetes among Hispanics/Latinos from diverse backgrounds: The Hispanic Community Health Study/Study of Latinos (HCHS/SOL). Diabetes Care. 2014;37(8):2233-9.

2. Siega-Riz AM, Sotres-Alvarez D, Ayala GX, Ginsberg M, Himes JH, Liu K, et al. Food-group and nutrient-density intakes by Hispanic and Latino backgrounds in the Hispanic Community Health Study/Study of Latinos. Am J Clin Nutr. 2014;99(6):1487-98.

3. Chiuve SE, Fung TT, Rimm EB, Hu FB, McCullough ML, Wang M, et al. Alternative dietary indices both strongly predict risk of chronic disease. J Nutr. 2012;142(6):1009-18.

4. Mattei J, Sotres-Alvarez D, Daviglus ML, Gallo LC, Gellman M, Hu FB, et al. Diet quality and its association with cardiometabolic risk factors vary by Hispanic and Latino ethnic background in the Hispanic Community Health Study/Study of Latinos. J Nutr. 2016;146(10):2035-44.

5. Global physical activity surveillance: World Health Organization; [Available from: <https://www.who.int/ncds/surveillance/steps/GPAQ/en/>.

6. Hoos T, Espinoza N, Marshall S, Arredondo EM. Validity of the global physical activity questionnaire (GPAQ) in adult Latinas. J Phys Act Health. 2012;9(5):698-705.

7. Bull FC, Maslin TS, Armstrong T. Global physical activity questionnaire (GPAQ): Nine country reliability and validity study. J Phys Act Health. 2009;6(6):790-804.

8. HCHS/SOL physical activity questionnaire 2011 [Available from: <https://sites.cscc.unc.edu/hchs/system/files/forms/UNLICOMMPhysicalActivityPAE02182008.pdf>.

9. Hispanic Community Health Study/Study of Latinos: Manuals and forms nd [Available from: <https://sites.cscc.unc.edu/hchs/manuals-forms>.

10. Lavange LM, Kalsbeek WD, Sorlie PD, Aviles-Santa LM, Kaplan RC, Barnhart J, et al. Sample design and cohort selection in the Hispanic Community Health Study/Study of Latinos. Ann Epidemiol. 2010;20(8):642-9.

11. HCHS/SOL analysis methods at baseline version 4. HCHS/SOL Coordinating Center; 2016.Available from: <https://sites.cscc.unc.edu/hchs/sites/default/files/public_docs/HCHS%20Analysis%20Methods%20V4.0%2020160927FINAL.pdf>.

12. HCHS/SOL analysis methods - visit 2. HCHS/SOL Coordinating Center; 2020.Available from: <https://sites.cscc.unc.edu/hchs/sites/default/files/public_docs/HCHS%20Visit%202%20Analysis%20Methods%20V2.0%20July%202020.pdf>.

13. HCHS/SOL physical activity data overview, methods and guidelines. HCHS/SOL Coordinating Center; 2014.Available from: <https://sites.cscc.unc.edu/hchs/sites/default/files/public_docs/HCHS%20Physical%20Activity%20Data%20Overview%20Methods%20Guidelines_V2_May2014_20140514final.pdf>.

14. Evenson KR, Sotres-Alvarez D, Deng YU, Marshall SJ, Isasi CR, Esliger DW, et al. Accelerometer adherence and performance in a cohort study of US Hispanic adults. Med Sci Sports Exerc. 2015;47(4):725-34.

**Supplemental Results**

Supplemental Table 1. Predicted incidence rates and hazard ratio estimates of incident diabetes per 1000 step increments, HCHS/SOL cohort (2008-2017).

| Steps/day | diabetes based on self-reported diagnosis, medications, labs  (3 criteria definition)  n=6,634 | | diabetes based on medications and labs  (2 criteria definition)  n=6,633 | |
| --- | --- | --- | --- | --- |
|  | adjusted IR per 10,000 p-years | adjusted HR (95% CI) | adjusted IR per 10,000 p-years | adjusted HR (95% CI) |
| 1,000 | 9.5 (2.1, 44.0) | 1.05(0.99,1.12) | 3.2 (0.5, 22.3) | 1.09(1.01,1.17) |
| 2,000 | 9.3 (2.0, 43.1) | 1.03(1.00,1.07) | 3.1 (0.5, 21.5) | 1.05(1.01,1.10) |
| 3,400 | 9.0 (1.9, 41.9) | ref | 3.0 (0.4, 20.5) | ref |
| 4,000 | 8.9 (1.9, 41.4) | 0.99(0.97,1.00) | 2.9 (0.4, 20.1) | 0.98(0.96,1.00) |
| 5,000 | 8.7 (1.9, 40.6) | 0.97(0.93,1.00) | 2.8 (0.4, 19.4) | 0.94(0.90,0.99) |
| 6,000 | 8.5 (1.8, 39.8) | 0.95(0.89,1.01) | 2.8 (0.4, 18.8) | **0.91(0.84,0.99)** |
| 7,000 | 8.4 (1.8, 39.0) | 0.92(0.85,1.01) | 2.7 (0.4, 18.2) | **0.88(0.79,0.99)** |
| 8,000 | 8.2 (1.8, 38.3) | 0.90(0.81,1.01) | 2.6 (0.4, 17.6) | **0.85(0.74,0.98)** |
| 9,000 | 8.0 (1.7, 37.6) | 0.89(0.77,1.02) | 2.5 (0.4, 17.0) | **0.82(0.69,0.98)** |
| 10,000 | 7.9 (1.7, 37.0) | 0.87(0.74,1.02) | 2.4 (0.4, 16.5) | **0.79(0.64,0.97)** |
| 11,000 | 7.7 (1.6, 36.3) | 0.85(0.70,1.02) | 2.3 (0.3, 16.0) | **0.76(0.60,0.97)** |
| 12,000 | 7.5 (1.6, 35.7) | 0.83(0.67,1.03) | 2.3 (0.3, 15.5) | **0.74(0.56,0.97)** |
| 13,000 | 7.4 (1.5, 35.1) | 0.81(0.64,1.03) | 2.2 (0.3, 15.0) | **0.71(0.53,0.96)** |
| 14,000 | 7.2 (1.5, 34.5) | 0.79(0.61,1.03) | 2.1 (0.3, 14.6) | **0.69(0.49,0.96)** |
| 15,000 | 7.1 (1.5, 34.0) | 0.78(0.58,1.04) | 2.1 (0.3, 14.2) | **0.66(0.46,0.95)** |
| 16,000 | 6.9 (1.4, 33.4) | 0.76(0.56,1.04) | 2.0 (0.3, 13.7) | **0.64(0.43,0.95)** |
| 17,000 | 6.8 (1.4, 32.9) | 0.74(0.53,1.04) | 1.9 (0.3, 13.3) | **0.62(0.40,0.95)** |
| 18,000 | 6.6 (1.4, 32.4) | 0.73(0.51,1.05) | 1.9 (0.3, 13.0) | **0.60(0.38,0.94)** |
| 19,000 | 6.5 (1.3, 32.0) | 0.71(0.48,1.05) | 1.8 (0.3, 12.6) | **0.58(0.35,0.94)** |
| 20,000 | 6.4 (1.3, 31.5) | 0.70(0.46,1.05) | 1.7 (0.2, 12.2) | **0.56(0.33,0.93)** |

Abbreviations: CI=confidence interval, HR= hazard ratio, IR=incidence rate, p-years= person-years

Models adjusted for age (continuous), quadratic term for age, sex (male, female), Latino background by HCHS/SOL field center (17 level categorical variable), education (< high school/no GED, high school/GED, > high school), married/partner status (yes, no), employment (yes, no), years in the US (born in US, < 10 years, > 10 years), self-rated general health (excellent/very good, good, fair/poor), mobility limitations (yes, no), cigarette pack years (continuous), alcoholic drinks per week (continuous), energy intake (continuous), AHEI-2010 (continuous) and accelerometer wear time (continuous, hours per day).

The referent of 3400 steps/day is the 10^th^ percentile of the steps/day distribution.

Supplemental Table 2. Associations of step volume and cadence with the risk of diabetes further adjusted for body mass index, HCHS/SOL cohort (2008-2017).

| Step metric - per day | diabetes based on self-reported diagnosis, medications, labs (3 criteria definition) n=6,634 | diabetes based on medications and labs (2 criteria definition) n=6,633 |
| --- | --- | --- |
|  | HR (95% CI) | HR (95% CI) |
| **Graduated step index, steps/day** | |  |
| < ,5000 | ref | ref |
| 5,000 - < 7,500 | 1.05(0.80,1.38) | 0.94(0.68,1.30) |
| 7,500 - < 10,000 | 1.21(0.91,1.60) | 1.26(0.88,1.81) |
| 10,000 - < 12,5000 | 0.95(0.65,1.38) | 0.75(0.51,1.10) |
| >12,500 | 0.97(0.68,1.38) | 0.91(0.59,1.40) |
| p for trend | 0.863 | 0.535 |
| per 1,000 steps | 0.99(0.97,1.02) | 0.99(0.96,1.02) |
|  | |  |
| **Peak 30 cadence, step/min** | |  |
| < 60 | ref | ref |
| 60 - < 80 | 1.02(0.78,1.31) | 1.07(0.77,1.47) |
| 80 - < 100 | 0.95(0.73,1.24) | 1.02(0.73,1.41) |
| >100 | 0.76(0.52,1.09) | 0.86(0.52,1.42) |
| p for trend | 0.162 | 0.627 |
| per 10 step/min increase | 0.99(0.95,1.03) | 0.99(0.93,1.05) |
|  | | |
| **Minutes spent at different cadence** | | |
| *> 40 steps/min, min per day* | |  |
| < 33 | ref | ref |
| 33 - < 55 | 1.14(0.87,1.50) | 1.15(0.84,1.59) |
| 55 - < 87 | 1.05(0.81,1.37) | 1.11(0.80,1.54) |
| >87 | 0.91(0.66,1.26) | 0.78(0.54,1.13) |
| p for trend | 0.559 | 0.251 |
| per 10 minutes | 0.99(0.97,1.01) | 0.99(0.96,1.02) |
|  | |  |
| *> 70 steps/min, min per day* | |  |
| < 10 | ref | ref |
| 10 - < 21 | 1.02(0.78,1.32) | 1.17(0.86,1.59) |
| 21 - < 39 | 1.10(0.85,1.43) | 1.13(0.83,1.52) |
| >39 | 0.84(0.62,1.14) | 0.90(0.62,1.31) |
| p for trend | 0.468 | 0.683 |
| per 10 minutes | 0.98(0.93,1.03) | 0.98(0.92,1.05) |
|  | |  |
| *> 100 steps/min, min per day* | |  |
| < 2 | ref | ref |
| 2 - < 7 | 1.05(0.80,1.37) | 1.03(0.75,1.40) |
| 7 - < 17 | 1.07(0.83,1.38) | 1.28(0.94,1.73) |
| >17 | 0.87(0.66,1.14) | 0.92(0.65,1.30) |
| p for trend | 0.403 | 0.872 |
| per 10 minutes | 0.98(0.91,1.06) | 1.00(0.90,1.11) |

Abbreviations: CI=confidence interval, HR= hazard ratio.

Models adjusted for age (continuous), quadratic term for age, sex (male, female), Latino background by HCHS/SOL field center (17 level categorical variable), education (< high school/no GED, high school/GED, > high school), married/partner status (yes, no), employment (yes, no), years in the US (born in US, < 10 years, > 10 years), self-rated general health (excellent/very good, good, fair/poor), mobility limitations (yes, no), cigarette pack years (continuous), alcoholic drinks per week (continuous), energy intake (continuous), AHEI-2010 (continuous) accelerometer wear time (continuous, hours per day), and BMI (continuous).

Supplemental Table 3. Hazard ratios and 95% CI of the association of steps/day with incident diabetes as defined by self-reported diagnosis, medications, and labs (3 criteria definition) by modifying factors, HCHS/SOL cohort (2008-2017).

| Modifier | 2,000 steps/day | 3,400 steps/day | 5,000 steps/day | 7,000 steps/per | 10,000 steps/day | 14,000 steps/day | LRT p-value |
| --- | --- | --- | --- | --- | --- | --- | --- |
| Age |  |  |  |  |  |  | 0.40 |
| < 50 | 1.02(0.98,1.07) | ref | 0.97(0.93,1.02) | 0.94(0.84,1.05) | 0.89(0.73,1.10) | 0.83(0.60,1.16) |  |
| > 50 | 1.04(0.99,1.10) | ref | 0.95(0.90,1.01) | 0.90(0.79,1.02) | 0.82(0.65,1.04) | 0.73(0.50,1.07) |  |
| Gender |  |  |  |  |  |  | 0.49 |
| Female | 1.04(0.99,1.09) | ref | 0.96(0.90,1.01) | 0.91(0.80,1.03) | 0.83(0.66,1.05) | 0.75(0.51,1.09) |  |
| Male | 1.02(0.98,1.07) | ref | 0.97(0.92,1.03) | 0.94(0.83,1.06) | 0.89(0.71,1.12) | 0.83(0.58,1.19) |  |
| Occupational physical activity | |  |  |  |  |  | 0.62 |
| No | 1.05(0.98,1.14) | ref | 0.94(0.86,1.03) | 0.87(0.71,1.06) | 0.78(0.54,1.12) | 0.67(0.37,1.20) |  |
| Yes | 1.04(0.99,1.09) | ref | 0.96(0.90,1.01) | 0.90(0.80,1.03) | 0.83(0.66,1.05) | 0.74(0.51,1.08) |  |
| Obesity |  |  |  |  |  |  | 0.02 |
| Not obese | 1.00(0.95,1.05) | ref | 1.00(0.95,1.06) | 1.01(0.89,1.14) | 1.01(0.81,1.27) | 1.02(0.71,1.46) |  |
| Obese | 1.05(1.00,1.09) | ref | 0.95(0.90,1.00) | 0.89(0.79,1.00) | 0.81(0.65,1.01) | 0.71(0.50,1.01) |  |
| Homa IR |  |  |  |  |  |  | 0.92 |
| Normal | 1.01(0.97,1.06) | ref | 0.99(0.94,1.04) | 0.97(0.87,1.09) | 0.95(0.77,1.17) | 0.93(0.66,1.29) |  |
| High | 1.01(0.96,1.06) | ref | 0.99(0.93,1.04) | 0.97(0.85,1.10) | 0.94(0.75,1.20) | 0.91(0.62,1.33) |  |
| Prediabetes | |  |  |  |  |  | 0.83 |
| Normal | 1.03(0.96,1.10) | ref | 0.97(0.90,1.05) | 0.93(0.78,1.11) | 0.87(0.63,1.20) | 0.80(0.48,1.35) |  |
| Prediabetes | 1.03(0.99,1.08) | ref | 0.96(0.92,1.01) | 0.92(0.83,1.02) | 0.85(0.70,1.03) | 0.77(0.57,1.05) |  |
| Hispanic/Latino heritage | |  |  |  |  |  | 0.62 |
| Central Amer. | 1.08(0.98,1.19) | ref | 0.92(0.82,1.03) | 0.82(0.63,1.06) | 0.70(0.43,1.12) | 0.56(0.26,1.19) |  |
| Cuban | 1.02(0.92,1.12) | ref | 0.98(0.88,1.10) | 0.96(0.75,1.23) | 0.93(0.59,1.46) | 0.89(0.43,1.84) |  |
| Dominican | 1.03(0.94,1.12) | ref | 0.97(0.88,1.07) | 0.93(0.75,1.16) | 0.88(0.59,1.31) | 0.82(0.43,1.54) |  |
| Mexican | 1.05(1.00,1.11) | ref | 0.94(0.89,1.00) | 0.88(0.76,1.01) | 0.79(0.61,1.01) | 0.68(0.45,1.02) |  |
| Puerto Rican | 1.01(0.95,1.07) | ref | 0.99(0.93,1.06) | 0.98(0.84,1.15) | 0.97(0.73,1.29) | 0.95(0.60,1.50) |  |
| South American | 1.04(0.89,1.22) | ref | 0.95(0.80,1.14) | 0.90(0.60,1.34) | 0.82(0.39,1.71) | 0.73(0.22,2.36) |  |
| Multi/Other | 1.01(0.89,1.14) | ref | 0.99(0.86,1.14) | 0.97(0.71,1.33) | 0.95(0.54,1.69) | 0.93(0.37,2.32) |  |

Predicted estimates at the 2^nd^ percentile (2,000 steps/day), 25^th^ percentile (5,000 steps/day), 50^th^ percentile (7,000 steps/day), 75^th^ percentile (10,000 steps/day), and 90^th^ percentile (14,000 steps/day). Within each stratum estimates compared to the 10^th^ percentile of steps/day (3,400 steps/day).

Abbreviations: Central Amer.= Central American, CI=confidence interval, LRT= likelihood ratio test, HR= hazard ratio, HOMA IR=homeostasis model assessment of insulin resistance

Models adjusted for age (continuous), quadratic term for age, sex (male, female), Latino background by HCHS/SOL field center (17 level categorical variable), education (< high school/no GED, high school/GED, > high school), married/partner status (yes, no), employment (yes, no), years in the US (born in US, < 10 years, > 10 years), self-rated general health (excellent/very good, good, fair/poor), mobility limitations (yes, no), cigarette pack years (continuous), alcoholic drinks per week (continuous), energy intake (continuous), AHEI-2010 (continuous) and accelerometer wear time (continuous, hours per day).

The model with occupational physical activity as a modifier is only among those who reported part- or full-time employment (n=3799) and does not include a covariate for employment, but otherwise is adjusted for the same covariates as other models.

The model with Hispanic/Latino heritage as a modifier is adjusted for HCHS/SOL field center (4 categories) rather than Latino background by HCHS/SOL field center (17 level categorical variable).

Supplemental Table 4. Incidence rates and 95% CI per 10,000 person-years of the association of steps/day with incident diabetes as defined by self-reported diagnosis, medications, and labs (3 criteria definition) by modifying factors, HCHS/SOL cohort (2008-2017).

| Modifier | 2,000 steps/day | 3,400 steps/day | 5,000 steps/day | 7,000 steps/per | 10,000 steps/day | 14,000 steps/day |
| --- | --- | --- | --- | --- | --- | --- |
| Age |  |  |  |  |  |  |
| < 50 | 9.1 (1.9, 43.1) | 8.8 (1.9, 41.9) | 8.5 (1.8, 40.5) | 8.2 (1.7, 39.0) | 7.8 (1.6, 36.9) | 7.2 (1.5, 34.6) |
| > 50 | 8.6 (1.7, 44.0) | 8.3 (1.6, 42.6) | 8.0 (1.6, 41.0) | 7.6 (1.5, 39.3) | 7.1 (1.4, 37.0) | 6.4 (1.2, 34.5) |
| Gender |  |  |  |  |  |  |
| Female | 9.6 (2.1, 44.9) | 9.2 (2.0, 43.0) | 8.8 (1.9, 41.1) | 8.3 (1.8, 38.8) | 7.6 (1.6, 35.9) | 6.8 (1.4, 32.6) |
| Male | 7.6 (1.5, 32.3) | 7.4 (1.5, 36.5) | 7.2 (1.5, 35.6) | 7.0 (1.4, 34.7) | 6.7 (1.3, 33.5) | 6.3 (1.2, 32.2) |
| Occupational physical activity | |  |  |  |  |  |
| No | 3.1 (0.3, 32.8) | 2.9 (0.3, 31.3) | 2.8 (0.3, 29.7) | 2.6 (0.2, 28.0) | 2.3 (0.2, 25.9) | 2.0 (0.2, 23.6) |
| Yes | 3.3 (0.3, 38.0) | 3.2 (0.3, 36.5) | 3.1 (0.3, 34.9) | 2.9 (0.3, 33.0) | 2.7 (0.2, 30.5) | 2.4 (0.2, 27.7) |
| Obesity |  |  |  |  |  |  |
| Not obese | 6.0 (1.3, 26.9) | 6.0 (1.3, 27.0) | 6.0 (1.3, 27.2) | 6.0 (1.3, 27.6) | 6.1 (1.3, 28.2) | 6.1 (1.3, 29.3) |
| Obesity | 14.7 (3.2, 68.1) | 14.1 (3.1, 65.0) | 13.4 (2.9, 61.6) | 12.6 (2.7, 57.8) | 11.4 (2.5, 52.8) | 10.1 (2.1, 47.1) |
| Homa IR |  |  |  |  |  |  |
| Normal | 5.6 (1.3, 23.8) | 5.5 (1.3, 23.6) | 5.4 (1.3, 23.4) | 5.3 (1.2, 23.2) | 5.2 (1.2, 23.0) | 5.0 (1.1, 22.9) |
| High | 14.6 (3.3, 64.2) | 14.5 (3.3, 63.4) | 14.4 (3.3, 62.6) | 14.2 (3.3, 61.7) | 14.0 (3.2, 60.8) | 13.7 (3.1, 60.1) |
| Prediabetes | |  |  |  |  |  |
| Normal | 10.6 (2.2, 49.9) | 10.2 (2.2, 47.9) | 9.8 (2.1, 45.9) | 9.3 (2.0, 43.8) | 8.6 (1.8, 41.1) | 7.8 (1.6, 38.6) |
| Prediabetes | 49.8 (11.0, 226.3) | 48.4 (10.6, 220.2) | 46.8 (10.3, 213.7) | 44.9 (9.8, 206.2) | 42.2 (9.1, 196.1) | 38.8 (8.2, 184.6) |
| Hispanic/Latino heritage | |  |  |  |  |  |
| Central American | 4.0 (0.9, 18.3) | 3.8 (0.8, 16.9) | 3.5 (0.8, 15.5) | 3.2 (0.7, 14.2) | 2.8 (0.6, 12.6) | 2.4 (0.5, 11.4) |
| Cuban | 3.5 (0.8, 15.2) | 3.4 (0.8, 15.0) | 3.4 (0.8, 14.9) | 3.3 (0.7, 14.9) | 3.3 (0.7, 15.2) | 3.2 (0.6, 16.1) |
| Dominican | 4.9 (0.9, 26.7) | 4.9 (0.9, 25.6) | 4.8 (0.9, 24.6) | 4.6 (0.9, 23.5) | 4.6 (0.9, 22.3) | 4.2 (0.8, 21.3) |
| Mexican | 7.5 (1.6, 34.6) | 7.1 (1.5, 32.6) | 6.6 (1.4, 30.6) | 6.1 (1.3, 28.3) | 5.4 (1.1, 25.3) | 4.6 (0.9, 22.1) |
| Puerto Rican | 5.6 (1.2, 25.9) | 5.6 (1.2, 25.8) | 5.6 (1.2, 25.8) | 5.6 (1.2, 25.9) | 5.6 (1.2, 26.3) | 5.7 (1.2, 27.1) |
| South American | 3.6 (0.6, 19.7) | 3.4 (0.7, 17.9) | 3.3 (0.7, 16.3) | 3.1 (0.6, 14.8) | 2.8 (0.6, 13.6) | 2.5 (0.5, 13.4) |
| Multi/Other | 7.4 (1.3, 41.9) | 7.3 (1.3, 39.7) | 7.2 (1.4, 37.6) | 7.0 (1.4, 35.6) | 6.8 (1.4, 34.0) | 6.5 (1.3, 33.8) |

Predicted estimates at the 2^nd^ percentile (2,000 steps/day), 25^th^ percentile (5,000 steps/day), 50^th^ percentile (7,000 steps/day), 75^th^ percentile (10,000 steps/day), and 90^th^ percentile (14,000 steps/day).

Abbreviations: CI=confidence interval, HOMA IR=homeostasis model assessment of insulin resistance

Models adjusted for age (continuous), quadratic term for age, sex (male, female), Latino background by HCHS/SOL field center (17 level categorical variable), education (< high school/no GED, high school/GED, > high school), married/partner status (yes, no), employment (yes, no), years in the US (born in US, < 10 years, > 10 years), self-rated general health (excellent/very good, good, fair/poor), mobility limitations (yes, no), cigarette pack years (continuous), alcoholic drinks per week (continuous), energy intake (continuous), AHEI-2010 (continuous) and accelerometer wear time (continuous, hours per day).

The model with occupational physical activity as a modifier is only among those who reported part- or full-time employment (n=3799) and does not include a covariate for employment, but otherwise is adjusted for the same covariates as other models.

The model with Hispanic/Latino heritage as a modifier is adjusted for HCHS/SOL field center (4 categories) rather than Latino background by HCHS/SOL field center (17 level categorical variable).

Supplemental Table 5. Hazard ratios and 95% CI of the association of steps/day with incident diabetes as defined by medications and labs (2 criteria definition) by modifying factors, HCHS/SOL cohort (2008-2017).

| Modifier | 2,000 steps/day | 3,400 steps/day | 5,000 steps/day | 7,000 steps/per | 10,000 steps/day | 14,000 steps/day | LRT p-value |
| --- | --- | --- | --- | --- | --- | --- | --- |
| Age |  |  |  |  |  |  | 0.05 |
| < 50 | 1.03(0.97,1.09) | ref | 0.97(0.90,1.03) | 0.93(0.80,1.08) | 0.87(0.66,1.14) | 0.80(0.51,1.24) |  |
| > 50 | 1.08(1.02,1.15) | ref | **0.91(0.85,0.98)** | **0.81(0.70,0.95)** | **0.69(0.52,0.90)** | **0.55(0.35,0.85)** |  |
| Sex |  |  |  |  |  |  | 0.03 |
| Female | 1.09(1.02,1.17) | ref | **0.91(0.84,0.98)** | **0.80(0.68,0.96)** | **0.67(0.49,0.92)** | **0.52(0.31,0.88)** |  |
| Male | 1.03(0.97,1.09) | ref | 0.97(0.91,1.03) | 0.93(0.81,1.08) | 0.88(0.68,1.15) | 0.82(0.53,1.25) |  |
| Occupational physical activity | |  |  |  |  |  | 0.69 |
| No | 1.07(0.97,1.18) | ref | 0.92(0.82,1.04) | 0.84(0.65,1.08) | 0.72(0.45,1.16) | 0.59(0.28,1.26) |  |
| Yes | 1.06(1.00,1.12) | ref | 0.94(0.88,1.00) | 0.87(0.75,1.01) | 0.77(0.59,1.01) | 0.66(0.43,1.02) |  |
| Obesity |  |  |  |  |  |  | 0.13 |
| Not obese | 1.01(0.95,1.08) | ref | 0.99(0.91,1.07) | 0.97(0.81,1.16) | 0.95(0.69,1.30) | 0.91(0.55,1.53) |  |
| Obese | 1.05(1.00,1.11) | ref | 0.94(0.89,1.00) | 0.88(0.77,1.01) | 0.79(0.62,1.02) | 0.69(0.46,1.03) |  |
| Homa IR |  |  |  |  |  |  | 0.25 |
| Normal | 1.04(0.97,1.10) | ref | 0.96(0.89,1.03) | 0.91(0.77,1.08) | 0.85(0.63,1.15) | 0.77(0.47,1.25) |  |
| High | 1.01(0.95,1.07) | ref | 0.99(0.93,1.06) | 0.99(0.85,1.15) | 0.98(0.74,1.28) | 0.96(0.62,1.49) |  |
| Prediabetes | |  |  |  |  |  | 0.04 |
| Normal | 0.99(0.91,1.07) | ref | 1.01(0.93,1.11) | 1.03(0.85,1.26) | 1.06(0.74,1.52) | 1.10(0.61,1.97) |  |
| Prediabetes | 1.07(1.01,1.12) | ref | **0.93(0.88,0.99)** | **0.85(0.74,0.97)** | **0.74(0.58,0.95)** | **0.62(0.42,0.92)** |  |
| Hispanic/Latino heritage | |  |  |  |  |  | 0.52 |
| Central Amer. | 1.12(0.99,1.26) | ref | 0.88(0.77,1.01) | 0.75(0.56,1.02) | 0.59(0.34,1.04) | 0.43(0.18,1.06) |  |
| Cuban | 1.09(0.94,1.26) | ref | 0.91(0.77,1.07) | 0.81(0.56,1.17) | 0.67(0.34,1.33) | 0.53(0.18,1.58) |  |
| Dominican | 1.01(0.91,1.12) | ref | 0.99(0.88,1.11) | 0.98(0.75,1.27) | 0.96(0.59,1.56) | 0.94(0.43,2.04) |  |
| Mexican | 1.05(0.98,1.13) | ref | 0.94(0.87,1.02) | 0.88(0.74,1.04) | 0.79(0.57,1.08) | 0.68(0.41,1.14) |  |
| Puerto Rican | 1.02(0.94,1.10) | ref | 0.98(0.89,1.07) | 0.95(0.77,1.16) | 0.91(0.63,1.31) | 0.86(0.47,1.55) |  |
| South American | 1.12(0.97,1.31) | ref | 0.87(0.74,1.04) | 0.74(0.50,1.09) | 0.58(0.28,1.18) | 0.41(0.13,1.30) |  |
| Multi/Other | 1.06(0.92,1.22) | ref | 0.94(0.80,1.10) | 0.87(0.61,1.25) | 0.77(0.40,1.50) | 0.66(0.23,1.92) |  |

Predicted estimates at the 2^nd^ percentile (2,000 steps/day), 25^th^ percentile (5,000 steps/day), 50^th^ percentile (7,000 steps/day), 75^th^ percentile (10,000 steps/day), and 90^th^ percentile (14,000 steps/day). Within each stratum estimates compared to the 10^th^ percentile of steps/day (3,400 steps/day).

Abbreviations: Central Amer.= Central American, CI=confidence interval, LRT= likelihood ratio test, HR= hazard ratio, HOMA IR=homeostasis model assessment of insulin resistance

Models adjusted for age (continuous), quadratic term for age, sex (male, female), Latino background by HCHS/SOL field center (17 level categorical variable), education (< high school/no GED, high school/GED, > high school), married/partner status (yes, no), employment (yes, no), years in the US (born in US, < 10 years, > 10 years), self-rated general health (excellent/very good, good, fair/poor), mobility limitations (yes, no), cigarette pack years (continuous), alcoholic drinks per week (continuous), energy intake (continuous), AHEI-2010 (continuous) and accelerometer wear time (continuous, hours per day).

The model with occupational physical activity as a modifier is only among those who reported part- or full-time employment (n=3799) and does not include a covariate for employment, but otherwise is adjusted for the same covariates as other models.

The model with Hispanic/Latino heritage as a modifier is adjusted for HCHS/SOL field center (4 categories) rather than Latino background by HCHS/SOL field center (17 level categorical variable).

Supplemental Table 6. Incidence rates and 95% CI per 10,000 person-years of the association of steps/day with incident diabetes as defined by medications and labs (2 criteria definition) by modifying factors, HCHS/SOL cohort (2008-2017).

| Modifier | 2,000 steps/day | 3,400 steps/day | 5,000 steps/day | 7,000 steps/per | 10,000 steps/day | 14,000 steps/day |
| --- | --- | --- | --- | --- | --- | --- |
| Age |  |  |  |  |  |  |
| < 50 | 2.9 (0.4, 20.5) | 2.8 (0.4, 19.7) | 2.7 (0.4, 18.9) | 2.6 (0.4, 17.9) | 2.4 (0.3, 16.7) | 2.2 (0.3, 15.3) |
| > 50 | 3.3 (0.4, 26.1) | 3.1 (0.4, 24.4) | 2.8 (0.4, 22.7) | 2.6 (0.3, 20.7) | 2.2 (0.3, 18.1) | 1.8 (0.2, 15.3) |
| Sex |  |  |  |  |  |  |
| Female | 3.6 (0.5, 25.3) | 3.3 (0.5, 22.9) | 3.0 (0.4, 20.5) | 2.6 (0.4, 17.8) | 2.2 (0.3, 14.7) | 1.7 (0.3, 11.5) |
| Male | 3.4 (0.5, 23.3) | 2.7 (0.4, 19.3) | 3.2 (0.5, 22.5) | 3.1 (0.4, 22.1) | 3.0 (0.4, 21.7) | 2.9 (0.4, 21.2) |
| Occupational physical activity | |  |  |  |  |  |
| No | 1.0 (0.05, 20.5) | 0.9 (0.04, 19.4) | 0.9 (0.04, 18.3) | 0.8 (0.04, 17.1) | 0.7 (0.03, 15.6) | 0.6 (0.03, 14.0) |
| Yes | 1.2 (0.06, 25.5) | 1.1 (0.05, 24.3) | 1.1 (0.05, 23.1) | 1.0 (0.04, 21.7) | 0.9 (0.04, 19.8) | 0.8 (0.04, 17.7) |
| Obesity |  |  |  |  |  |  |
| Not obese | 1.9 (0.3, 12.5) | 1.9 (0.3, 12.3) | 1.9 (0.3, 12.2) | 1.9 (0.3, 12.1) | 1.8 (0.3, 12.1) | 1.8 (0.3, 12.3) |
| Obese | 6.0 (0.9, 40.2) | 5.6 (0.8, 37.9) | 5.3 (0.8, 35.4) | 4.9 (0.7, 32.7) | 4.4 (0.7, 29.1) | 3.8 (0.6, 25.0) |
| Homa IR |  |  |  |  |  |  |
| Normal | 1.8 (0.3, 11.3) | 1.8 (0.3, 10.8) | 1.7 (0.3, 10.4) | 1.7 (0.3, 9.9) | 1.6 (0.3, 9.2) | 1.4 (0.2, 8.5) |
| High | 5.9 (1.0, 35.7) | 5.9 (1.0, 35.3) | 5.8 (1.0, 35.0) | 5.7 (1.0, 34.5) | 5.6 (0.9, 34.1) | 5.5 (0.9, 33.7) |
| Prediabetes | |  |  |  |  |  |
| Normal | 2.8 (0.4, 19.5) | 2.8 (0.4, 19.4) | 2.8 (0.4, 19.4) | 2.8 (0.4, 19.4) | 2.8 (0.4, 19.8) | 2.9 (0.4, 20.7) |
| Prediabetes | 29.4 (4.4, 197.7) | 27.8 (4.1, 186.4) | 26.1 (3.9, 174.5) | 24.1 (3.6, 161.0) | 21.4 (3.2, 143.2) | 18.3 (2.7, 123.4) |
| Hispanic/Latino heritage | |  |  |  |  |  |
| Central American | 2.3 (0.4, 15.1) | 2.1 (0.3, 13.7) | 2.0 (0.3, 12.3) | 1.8 (0.3, 11.0) | 1.5 (0.2, 9.5) | 1.2 (0.2, 8.2) |
| Cuban | 2.0 (0.3, 12.5) | 1.9 (0.3, 11.4) | 1.7 (0.3, 10.4) | 1.6 (0.3, 9.3) | 1.3 (0.2, 8.1) | 1.1 (0.2, 7.0) |
| Dominican | 2.5 (0.3, 21.6) | 2.5 (0.3, 20.9) | 2.5 (0.3, 20.1) | 2.5 (0.3, 19.4) | 2.5 (0.3, 18.6) | 2.4 (0.3, 18.2) |
| Mexican | 3.3 (0.5, 21.4) | 3.1 (0.5, 20.2) | 2.9 (0.4, 18.9) | 2.7 (0.4, 17.4) | 2.4 (0.4, 15.6) | 2.0 (0.3, 13.7) |
| Puerto Rican | 2.2 (0.3, 14.5) | 2.2 (0.3, 14.2) | 2.2 (0.3, 14.0) | 2.1 (0.3, 13.7) | 2.1 (0.3, 13.5) | 2.0 (0.3, 13.3) |
| South American | 2.3 (0.3, 18.6) | 2.0 (0.3, 15.1) | 1.7 (0.2, 12.1) | 1.3 (0.2, 9.3) | 0.9 (0.1, 6.5) | 0.6 (0.1, 4.4) |
| Multi/Other | 3.4 (0.4, 27.8) | 3.4 (0.4, 26.2) | 3.3 (0.4, 24.8) | 3.2 (0.4, 23.6) | 3.1 (0.4, 22.8) | 2.9 (0.4, 23.3) |

Predicted estimates at the 2^nd^ percentile (2,000 steps/day), 25^th^ percentile (5,000 steps/day), 50^th^ percentile (7,000 steps/day), 75^th^ percentile (10,000 steps/day), and 90^th^ percentile (14,000 steps/day).

Abbreviations: CI=confidence interval, HOMA IR=homeostasis model assessment of insulin resistance

Models adjusted for age (continuous), quadratic term for age, sex (male, female), Latino background by HCHS/SOL field center (17 level categorical variable), education (< high school/no GED, high school/GED, > high school), married/partner status (yes, no), employment (yes, no), years in the US (born in US, < 10 years, > 10 years), self-rated general health (excellent/very good, good, fair/poor), mobility limitations (yes, no), cigarette pack years (continuous), alcoholic drinks per week (continuous), energy intake (continuous), AHEI-2010 (continuous) and accelerometer wear time (continuous, hours per day).

The model with occupational physical activity as a modifier is only among those who reported part- or full-time employment (n=3799) and does not include a covariate for employment, but otherwise is adjusted for the same covariates as other models.

The model with Hispanic/Latino heritage as a modifier is adjusted for HCHS/SOL field center (4 categories) rather than Latino background by HCHS/SOL field center (17 level categorical variable).

Supplemental Table 7. The association of bouted stepping with incident diabetes, HCHS/SOL cohort (2008-2017).

| Step metric - per day | diabetes based on self-reported diagnosis, medications, labs  (3 criteria definition) n=6,634 | | diabetes based on medications and labs (2 criteria definition) n=6,633 | |  |  |
| --- | --- | --- | --- | --- | --- | --- |
|  | adjusted IR per 10,000 p-years | adjusted HR (95% CI) | adjusted IR per 10,000 p-years | adjusted HR (95% CI) |  |  |
| **Minutes spent in bouts at different cadence** | | | | |  |  |
| *> 40 steps/min, min per day* | | | |  |  |  |
| no bouts | 9.6 (2.1, 44.1) | ref | 3.2 (0.5, 22.6) | ref |  |  |
| 0 - < 11 | 9.7 (2.0, 46.4) | 1.01(0.74,1.37) | 3.5 (0.5, 24.3) | 1.05(0.74,1.49) |  |  |
| 11 - < 30 | 8.8 (1.9, 40.0) | 0.90(0.67,1.19) | 3.5 (0.5, 23.5) | 1.04(0.75,1.42) |  |  |
| >30 | 7.4 (1.5, 36.1) | 0.76(0.55,1.06) | 2.6 (0.4, 18.6) | 0.79(0.55,1.13) |  |  |
| p for trend |  | 0.050 |  | 0.161 |  |  |
|  | | | |  |  |  |
| *> 70 steps/min, min per day* | | | |  |  |  |
| no bouts | 9.9 (2.2, 45.8) | ref | 3.5 (0.5, 24.3) | ref |  |  |
| 0 - < 7 | 10.3 (2.1, 49.7) | 1.03(0.80,1.33) | 4.0 (0.6, 29.0) | 1.13(0.84,1.51) |  |  |
| 7 - < 17 | 9.0 (1.9, 42.2) | 0.90(0.69,1.15) | 3.6 (0.5, 26.0) | 1.04(0.77,1.41) |  |  |
| >17 | 7.4 (1.6, 34.9) | 0.77(0.59,1.01) | 2.8 (0.4, 19.7) | 0.89(0.65,1.23) |  |  |
| p for trend |  | 0.051 |  | 0.567 |  |  |
|  | | | |  |  |  |
| *> 100 steps/min, min per day* | | | |  |  |  |
| no bouts | 9.8 (2.1, 45.4) | ref | 3.5 (0.5, 23.9) | ref |  |  |
| 0 - < 5 | 8.9 (1.8, 42.9) | 0.86(0.64,1.16) | 3.7 (0.5, 26.6) | 1.02(0.74,1.42) |  |  |
| 5 - < 12 | 8.5 (1.8, 40.3) | 0.89(0.65,1.22) | 3.3 (0.5, 24.0) | 1.02(0.70,1.49) |  |  |
| >12 | 8.3 (1.7, 40.0) | 0.87(0.65,1.16) | 2.9 (0.4, 21.1) | 0.93(0.62,1.38) |  |  |
| p for trend |  | 0.251 |  | 0.807 |  |  |

Abbreviations: CI=confidence interval, HR= hazard ratio, IR=incidence rate, p-years= person-years

Models adjusted for age (continuous), quadratic term for age, sex (male, female), Latino background by HCHS/SOL field center (17 level categorical variable), education (< high school/no GED, high school/GED, > high school), married/partner status (yes, no), employment (yes, no), years in the US (born in US, < 10 years, > 10 years), self-rated general health (excellent/very good, good, fair/poor), mobility limitations (yes, no), cigarette pack years (continuous), alcoholic drinks per week (continuous), energy intake (continuous), AHEI-2010 (continuous) and accelerometer wear time (continuous, hours per day).

Supplemental Table 8. The association of percent of intense steps (> 100 steps/min) with incident diabetes adjusted for steps/day, HCHS/SOL cohort (2008-2017).

| Steps per day | Percent of intense steps (> 100 steps/min) | | | |
| --- | --- | --- | --- | --- |
|  | 1% | 10% | 20% | 30% |
| diabetes based on self-reported diagnosis, medications, labs (3 criteria definition) n=6,634 | | | | |
| 2,000 | 1.02(0.98,1.06) | 0.98(0.89,1.09) | 0.94(0.78,1.12) | 0.90(0.69,1.17) |
| 3,400 | ref | 0.96(0.89,1.03) | 0.92(0.78,1.07) | 0.87(0.68,1.12) |
| 5,000 | 0.97(0.93,1.01) | 0.93(0.87,1.00) | 0.89(0.77,1.03) | 0.85(0.68,1.07) |
| 7,000 | 0.93(0.85,1.02) | **0.90(0.82,0.99)** | **0.86(0.74,0.99)** | 0.82(0.66,1.02) |
| 10,000 | 0.88(0.74,1.05) | 0.85(0.73,1.00) | **0.81(0.68,0.97)** | **0.78(0.62,0.97)** |
| 14,000 | 0.82(0.62,1.09) | 0.79(0.61,1.03) | **0.76(0.58,0.98)** | **0.72(0.55,0.95)** |
| diabetes based on medications and labs (2 criteria definition) n=6,633 | | | | |
| 2,000 | 1.04(0.99,1.10) | 1.02(0.91,1.15) | 0.99(0.80,1.24) | 0.97(0.70,1.34) |
| 3,400 | ref | 0.97(0.89,1.07) | 0.95(0.78,1.16) | 0.92(0.68,1.25) |
| 5,000 | **0.95(0.90,0.99)** | 0.92(0.84,1.01) | 0.90(0.74,1.09) | 0.87(0.65,1.17) |
| 7,000 | **0.88(0.79,0.99)** | **0.86(0.76,0.98)** | 0.84(0.69,1.02) | 0.82(0.61,1.09) |
| 10,000 | **0.80(0.65,0.99)** | **0.78(0.64,0.96)** | **0.76(0.60,0.97)** | 0.74(0.54,1.01) |
| 14,000 | **0.70(0.49,0.99)** | **0.68(0.49,0.95)** | **0.66(0.47,0.93)** | **0.65(0.44,0.94)** |

Models adjusted for age (continuous), quadratic term for age, sex (male, female), Latino background by HCHS/SOL field center (17 level categorical variable), education (< high school/no GED, high school/GED, > high school), married/partner status (yes, no), employment (yes, no), years in the US (born in US, < 10 years, > 10 years), self-rated general health (excellent/very good, good, fair/poor), mobility limitations (yes, no), cigarette pack years (continuous), alcoholic drinks per week (continuous), energy intake (continuous), AHEI-2010 (continuous), accelerometer wear time (continuous, hours per day), and steps/day (continuous).

Supplemental Table 9. Pearson correlation coefficients between step metrics, average counts per minute, and minutes spent in moderate-to-vigorous physical activity, HCHS/SOL cohort (2008-2017).

|  | Steps/day | Average CPM | Min MVPA | Min > 40 steps/min | Min > 70 steps/min | Min > 100 steps/min | Peak 30 cadence |
| --- | --- | --- | --- | --- | --- | --- | --- |
| Steps/day | 1.00 | 0.69 | 0.66 | 0.97 | 0.78 | 0.55 | 0.72 |
| Average CPM | 0.69 | 1.00 | 0.87 | 0.71 | 0.61 | 0.47 | 0.54 |
| Min MVPA | 0.66 | 0.87 | 1.00 | 0.68 | 0.77 | 0.67 | 0.64 |
| Min > 40 steps/min | 0.97 | 0.71 | 0.68 | 1.00 | 0.80 | 0.51 | 0.69 |
| Min > 70 steps/min | 0.78 | 0.61 | 0.77 | 0.80 | 1.00 | 0.87 | 0.83 |
| Min > 100 steps/min | 0.55 | 0.47 | 0.67 | 0.51 | 0.87 | 1.00 | 0.78 |
| Peak 30 cadence | 0.72 | 0.54 | 0.64 | 0.69 | 0.83 | 0.78 | 1.00 |

Abbreviations: CPM = counts per minute, Min = minutes, MVPA = moderate-to-vigorous physical activity
